# SK3/TRPC1/Orai1 complex regulates SOCE-dependent colon cancer cell migration: a novel opportunity to modulate anti-EGFR mAb action by the alkyl-lipid Ohmline

**DOI:** 10.18632/oncotarget.8786

**Published:** 2016-04-18

**Authors:** Maxime Guéguinou, Thomas Harnois, David Crottes, Arnaud Uguen, Nadine Deliot, Audrey Gambade, Aurélie Chantôme, Jean Pierre Haelters, Paul Alain Jaffrès, Marie Lise Jourdan, Günther Weber, Olivier Soriani, Philippe Bougnoux, Olivier Mignen, Nicolas Bourmeyster, Bruno Constantin, Thierry Lecomte, Christophe Vandier, Marie Potier-Cartereau

**Affiliations:** ^1^ INSERM UMR 1069, Université de Tours, Tours, France; ^2^ Equipe ERL 7368, CNRS, Université de Poitiers, Poitiers, France; ^3^ Department of Physiology, University of California, San Francisco, San Francisco, CA, USA; ^4^ CNRS-UMR 6521-Université de Brest, Brest, France; ^5^ GICC-UMR 7292 Université de Tours, Tours, France; ^6^ INSERM-UMR 1078 Université de Brest, Brest, France; ^7^ CHRU Brest, Service d'Anatomie et Cytologie Pathologiques, Brest, France; ^8^ CNRS UMR 7299, INSERM-UMR 1099, Université de Nice Sophia-Antipolis, Nice, France; ^9^ Ion Channels Network and Cancer-Cancéropôle Grand Ouest (IC-CGO), France; ^10^ CHRU Tours, Tours, France

**Keywords:** SOCE, Ohmline, lipid-raft channel complex, anti-EGFR mAbs, Akt signaling

## Abstract

**Background:**

Barely 10-20% of patients with metastatic colorectal cancer (mCRC) receive a clinical benefit from the use of anti-EGFR monoclonal antibodies (mAbs). We hypothesized that this could depends on their efficiency to reduce Store Operated Calcium Entry (SOCE) that are known to enhance cancer cells.

**Results:**

In the present study, we demonstrate that SOCE promotes migration of colon cancer cell following the formation of a lipid raft ion channel complex composed of TRPC1/Orai1 and SK3 channels. Formation of this complex is stimulated by the phosphorylation of the reticular protein STIM1 by EGF and activation of the Akt pathway. Our data show that, in a positive feedback loop SOCE activates both Akt pathway and SK3 channel activity which lead to SOCE amplification. This amplification occurs through the activation of Rac1/Calpain mediated by Akt. We also show that Anti-EGFR mAbs can modulate SOCE and cancer cell migration through the Akt pathway. Interestingly, the alkyl-lipid Ohmline, which we previously showed to be an inhibitor of SK3 channel, can dissociated the lipid raft ion channel complex through decreased phosphorylation of Akt and modulation of mAbs action.

**Conclusions:**

This study demonstrates that the inhibition of the SOCE-dependent colon cancer cell migration trough SK3/TRPC1/Orai1 channel complex by the alkyl-lipid Ohmline may be a novel strategy to modulate Anti-EGFR mAb action in mCRC.

## INTRODUCTION

Colorectal cancer is a major cause of morbidity and mortality throughout the world [1, 2]. The therapeutic arsenal available to treat colorectal cancer has recently been reinforced with two anti-EGFR monoclonal antibodies (mAbs i.e., cetuximab and panitumumab) [3, 4]. Nevertheless, only 10-20% of patients with metastatic colorectal cancer (mCRC) benefit from anti-EGFR mAbs therapy. These restricted benefits may be due to i) a constitutive activation of the PI3K-Akt pathway, mostly as consequence of K-Ras mutation [5]; ii) an abnormal PI3K status. It is therefore necessary to identify predictive biomarkers with expression may be associated with the efficiency of this biomedicine in order to optimize its use. Our studies have shown that regulation and activation of K^+^ and Ca^2+^ channels, involved in cell migration, are partly correlated to the activation of the EGFR pathway [6–9]. In non-excitable cells, Ca^2+^ homeostasis is regulated by voltage–independent Ca^2+^ channels, such as the Transient Receptor Potential (TRP) or Orai channels. During the last decade, these channels have been found to be expressed in various tumors types [10], and they can be involved in response of cancer cells to extracellular factors. Store operated Ca^2+^ entry (SOCE), is an ubiquitous Ca^2+^ entry pathway that is mediated by the interaction of a Ca^2+^ sensor at the endoplasmic reticulum (STIM1) and the plasma membrane channels Orai1. To reduce energy consumption and for a tighter regulation of their activities, Ca^2+^ channels appear to be associated with Ca^2+^-activated K^+^ channels to form complexes [11]. In tumor cells these complexes contribute to several cancer associated functions such as cell proliferation, cell migration and the capacity to develop metastases [11, 12]. We have proposed that theses complexes are spatially segregated in nanodomains such as lipid-rafts [13]. This particular subcellular localization has been observed in the case of the TRPC1 channelosome [14], and many ion channels have been found to be associated with in lipid-rafts [15]. We have recently discovered that a channel complex, formed by SK3 and Orai1, can localize exclusively in to lipid-rafts and controls bone metastasis development in a mouse orthotopic xenografted model of breast cancer [12]. In the present study, we used HCT-116 colon cancer cell line (K-Ras, PIK3CA mutated) to explore a functional presence of a K^+^/Ca^2+^ channel complex and to define the potential role of this channel complex in calcium and EGF–dependent cancer cell migration. Furthermore we assessed the role of the alkyl-lipid Ohmline, previously identified through its SK3/Orai1 complex inhibition properties [12], to modify the actions of anti-EGFR mAbs such as cetuximab and panitumumab currently used in colon cancer therapeutic.

## RESULTS

### The SK3 channel promotes cell migration of HCT-116 cells in an SOCE-dependent manner

**Figure 1:**
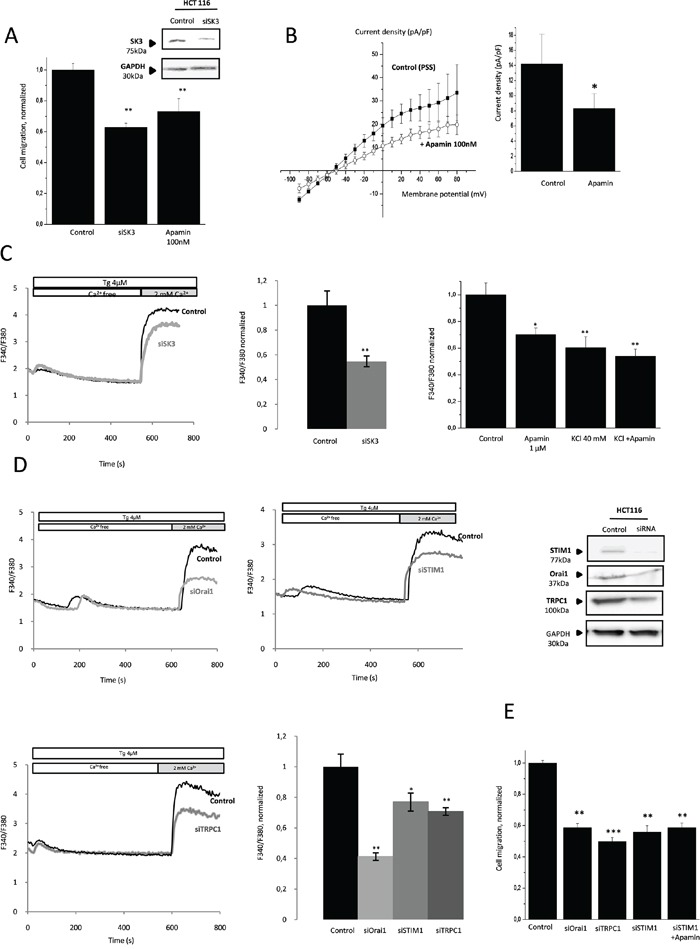
Migration of colon cancer cells HCT-116 is dependent on calcium-activated potassium channel SK3 and SOCE **A.** SK3 channel is involved in HCT-116 cell migration. Histograms showing HCT-116 cell migration transfected for 48h with siSK3 or treated with Apamin. The normalized cell number corresponds to the ratio of total number of migrating cells in presence of drugs/total number of migrating cells in control experiments. Results are expressed as mean ± SEM. **p<0.01, sample significantly different from control (N=3, n=9, Kruskal-Wallis test). Inset, Validation of SK3 protein extinction was performed by immunoblots 48h after transfection. **B.** SK3 current density-voltage relation obtained in control condition or after acute treatment of Apamin. The current density-voltage relationship was obtained by dividing the averaged steady-state currents elicited between - 90mV to + 80mV by the respective cell capacitance. Results are expressed as mean ± SEM. *p=0.05, sample significantly different from control (N=4, Mann–Whitney test). **C.** Left panel, silencing of SK3 decreases SOCE in HCT-116 cell migration. Fluorescence measurement and relative fluorescence of Ca^2+^entry after intracellular calcium store depletion by Tg in cells transfected with siSK3 or control siRNA. Data are means ± SEM. **p<0.01, sample significantly different from control (N=7, Mann–Whitney test). Right panel, plasma membrane depolarization induces a decrease of SOCE in HCT-116 cells. Histograms showing relative fluorescence of Ca^2+^entry after intracellular calcium store depletion by Tg in HCT-116 cells in control condition or after an acute treatment with 1μM Apamin or KCl 40 mM. Data represent means ± SEM. *p<0.05, **p<0.01, sample significantly different from control (N=3, Kruskal-Wallis test) **D.** Orai1, TRPC1 and STIM1 promote a store operated Ca^2+^ influx. Fluorescence measurement and relative fluorescence of Ca^2+^entry after intracellular calcium store depletion by Tg in transfected cells for 48h with siOrai1, siTRPC1 and siSTIM1. Data represent means ± SEM. *p<0.05, **p<0.01, sample significantly different from control (N=6, Kruskal-Wallis test). Inset, validation of Orai1, TRPC1 and STIM1 protein extinction by siRNA was performed by immunoblots 48h after transfection. **E.** Calcium channels Orai1 and TRPC1 and the protein STIM1 are involved in HCT-116 migration without additive effect of apamin treatment Data represent means ± SEM. **p<0.01, ***p<0.001, sample significantly different from control (N=6, Kruskal-Wallis test).

To investigate the role of SK3 in the migratory ability of the colon cancer cell HCT-116 line, we tested the effect of SK3-siRNA and Apamin on cell migration. As shown in Figure 1A, down-regulation of SK3 in cells treated with SK3-siRNA or Apamin decreased the number of migrating cells without affecting their viability (Supplementary Figure 1A). Correspondingly, Apamin decreased the current density-voltage relationship of the SK3 channel (Figure 1B). As displayed in Figure 1C, downregulation of SK3 expression by siRNA was associated with a decrease of SOCE. Moreover, depolarization of the plasma membrane induced by an acute application of Apamin or by KCl 40 mM, decreased SOCE without additive effect when we associated Apamin and KCl 40 mM. This suggests that, SK3 channel enhances SOCE by controlling the resting plasma membrane potential (Figure 1C, right). The protein STIM1 andtheTRPC1 and Orai1 channels have been shown to be Store-Operated Channels (SOC) regulating cancer cell migration [10]. Consequently, silencing STIM1, Orai1 and TRPC1 led to a decreased of SOCE evoked by the chemical SOCE inducer Thapsigargin (Tg) in HCT-116 cells (Figure 1D) without affecting cell proliferation (Supplementary Figure 1A). Furthermore, cell migration decreased (Figure 1E) without additive effect when a co-treatment siSTIM1/Apamin was performed (Figure 1E). Taken together, these data suggest that SOCE induced by STIM1/Orai1/TRPC1 plays a role in SK3-dependent colon cancer cell migration.

### STIM1 activation recruits an Orai1/TRPC1 complex into lipid rafts containing SK3 channels

**Figure 2:**
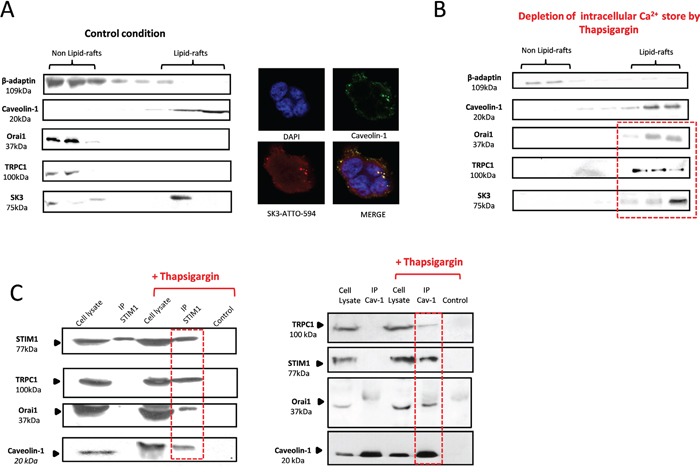
STIM1 activation, triggered by Ca2+ store depletion, recruits an Orai1/TRPC1 complex into lipid-rafts containing SK3 channels **A.** Left panel, Immunoblots representing membrane fractionation, on a sucrose gradient of cell lysate in control condition. SK3 is exclusively located into lipid-rafts whereas calcium channels Orai1 and TRPC1 are found outside lipid-rafts. Right panel, representative confocal images of SK3 and Caveolin-1 staining in HCT-116 cells showing immunocolocalization of SK3 and Caveolin-1. **B.** Depletion of intracellular calcium store by Tg induces the translocation of calcium channels Orai1 and TRPC1 into lipid-rafts whereas SK3 is always in lipid-rafts. Immunoblots representing membrane fractionation, on a sucrose gradient, of cells treated with 5μM Tg for 20 min. **C.** TRPC1, Orai1, STIM1 and caveolin-1 form a lipid-raft complex in HCT-116 after Ca^2+^ store depletion. Immunoblots depict co-immunoprecipitation between STIM1, TRPC1, Orai1 and Caveolin-1 before and after Ca^2+^ store depletion.

SK3 has been found to be localized exclusively in caveolae-lipid-rafts, whereas Orai1 was found outside (Figure 2A). Interestingly, depletion of Ca^2+^ store by Tg induced a recruitment of Orai1-TRPC1 channels into lipid-rafts (Figure 2B). Thus, depletion of reticularCa^2+^ store and consecutive activation of STIM1 may be required for the recruitment of Orai1-TRPC1 channels into lipid-rafts, potentially in the same membrane domain than SK3. To confirm this hypothesis, we performed co-immunoprecipitation experiments with STIM1 prior to- and after SOCE evoked by Tg. As shown in Figure 2C (left panel), STIM1 did not co-immunoprecipitate with neither Orai1 nor TRPC1 in control conditions. However, Tg treatment led to the co-immunoprecipitation of STIM1 with Orai1, TRPC1 and caveolin-1. The same results were obtained when caveolin-1 was co-immunoprecipitated with STIM1, TRPC1 and Orai1 (Figure 2C, right panel). This data suggest that Ca^2+^ stores depletion in these colon cancer cells is associated with a STIM1-dependent functional recruitment of Orai1-TRPC1 into lipid-rafts, thereby forming a complex with STIM1 and caveolin-1. This complex may interact with the lipid-raft and may form a Ca^2+^microdomain stimulating colon cancer cell migration in an SK3-dependent manner.

### Rac1 basal activity promotes SOCE-dependent migration through activation of the downstream effectors PI3K/Akt

**Figure 3:**
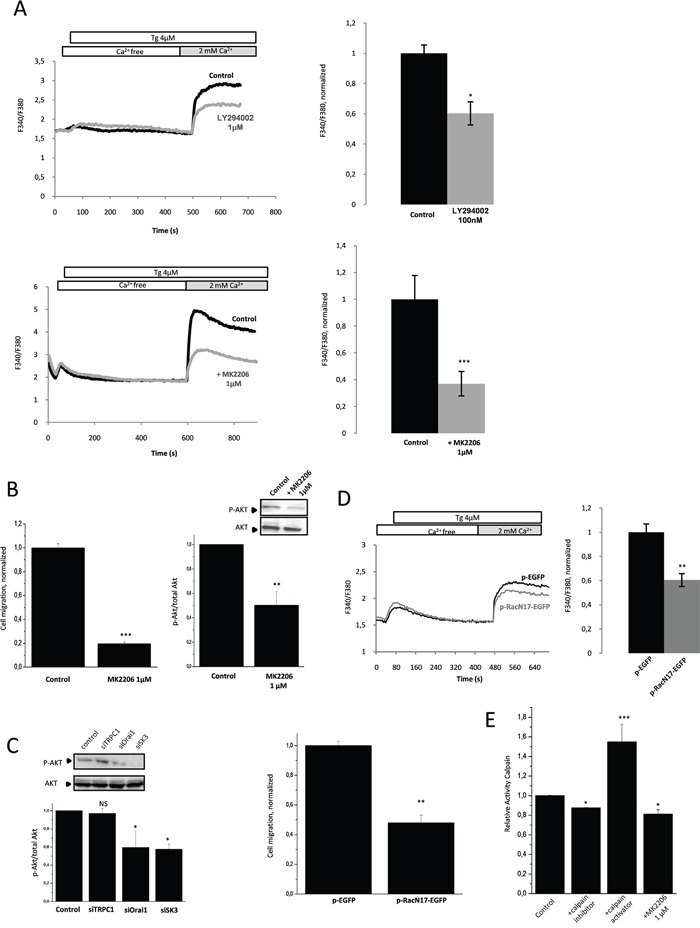
Rac1 basal activity promotes SOCE-dependent migration through the activation of the downstream effectors PI3K/Akt **A.** PI3K and Akt are involved in SOCE. Fluorescence measurement and relative fluorescence of Ca^2+^entry after intracellular calcium store depletion by Tg in cells treated for 1h with LY294002, a potent inhibitor of PI3K (upper panel,) or with MK2206, an inhibitor of Akt (lower panel). Data represent means ± SEM. *p<0.05 and ***p<0.001, sample significantly different from control (N=8, Mann–Whitney test). **B.** Left panel, Akt is involved in HCT-116 cell migration. Histograms showing cell migration with or without treatment by MK2206. Data are means ± SEM. ***p<0.001, sample significantly different from control (N=4, Mann–Whitney test). Right panel, phosphorylated Akt (P-Akt) levels (standardized based on total Akt) was determined by densitometry scanning to generate the values shown in the bar graph. Results are expressed as mean ± SEM. **p<0.01, sample significantly different from control (N=3, Kruskal-Wallis test). Inset, validation of P-Akt extinction by immunoblots after treatment with MK2206. **C.** Silencing of Orai1 and SK3 channels decrease Akt activation whereas siTRPC1 did not affect P-Akt. P-Akt levels (standardized based on total Akt) was determined by densitometry scanning to generate the values shown in the bar graph. Results are expressed as mean ± SEM. *p<0.05, sample significantly different from control (N=3, Kruskal-Wallis test). Immunoblots representing P-Akt and total Akt in cells under control condition and after transfection with siRNA for 24h. **D.** Inhibition of Rac1 by a dominant-negative form of Rac1(pRacN17) decreases SOCE (upper panel) and migration (lower panel) of HCT-116 cells. Fluorescence measurement and relative fluorescence of Ca^2+^entry after intracellular calcium store depletion by Tg in cells transfected with pRacN17and compared to a control condition (pGFP). Lower panel, histograms showing migration of HCT-116 cells transfected with P-RacN17-GFP plasmid or P-GFP plasmid after 24h. Data are means ± SEM. **p<0.01, sample significantly different from control (N=3, Mann–Whitney test). **E.** Calpain activity in HCT-116 cells. Inhibition of Akt by MK2206 decreases calpain activity. Results are expressed as mean ± SEM. *p<0.05 or ***p<0.001, sample significantly different from control (N=4, Kruskal-Wallis test).

HCT-116 cancer cells are characterized by a constitutive activation of the PI3K/Akt signaling pathway through activating mutations in PIK3CA. As shown in Figure 3A, pharmacological inhibition of PI3K/Akt pathway by LY294002 (inhibits PI3K activity via a competitive inhibition of an ATP binding site on the p85 α subunit) or MK2206 (inhibits auto-phosphorylation of both Akt T^308^ and S^473^ and prevents Akt-mediated phosphorylation of downstream signaling molecules) led to a decreased of SOCE. Furthermore, inhibition of Akt phosphorylation by MK-2206, confirmed by western blotting (Figure 3B, Inset), significantly decreased cell migration (Figure 3B). This data suggest that endogenous activation of the PI3K/Akt pathway may stimulate the SOCE-dependent cancer cell migration. Similary, SK3 and Orai1 but not TRPC1 down regulation, led to a decrease of Akt phosphorylation (Figure 3C). Thus, endogenous activation of the PI3K/Akt pathway is associated with a SOCE-dependent cell migration through formation of the TRPC1/Orai1/SK3 channel complex. Furthermore, we hypothesized that the constitutive activation of PI3K/Akt could modulate the small GTP binding protein Rac1 during the process of controlling SOCE-dependent cell migration. As shown in Figure 3D, expression of a dominant-negative form of Rac1 reduced SOCE and cell migration. Thus, Rac1 basal activity appears to promote SOCE-dependent migration, which is also mediated by activation of the PI3K/Akt pathway. We have previously shown that calpain causes a Ca^2+^-dependent proteolysis upstream of Rac1, and therefore regulates Ca^2+^ dependent cell migration [12]. We show here that basal activity of calpain could be down regulated by pharmacological inhibition of Akt, in accordance to calpain activity measured in HCT-116 cancer cells (Figure 3E). This data suggests that calpain may play an important role in the SOCE-dependent PI3K/Akt/Rac1 pathway.

### PI3K/Akt/Rac1 pathway mediates EGF-induced SOCE dependent cell migration by P-STIM1

**Figure 4:**
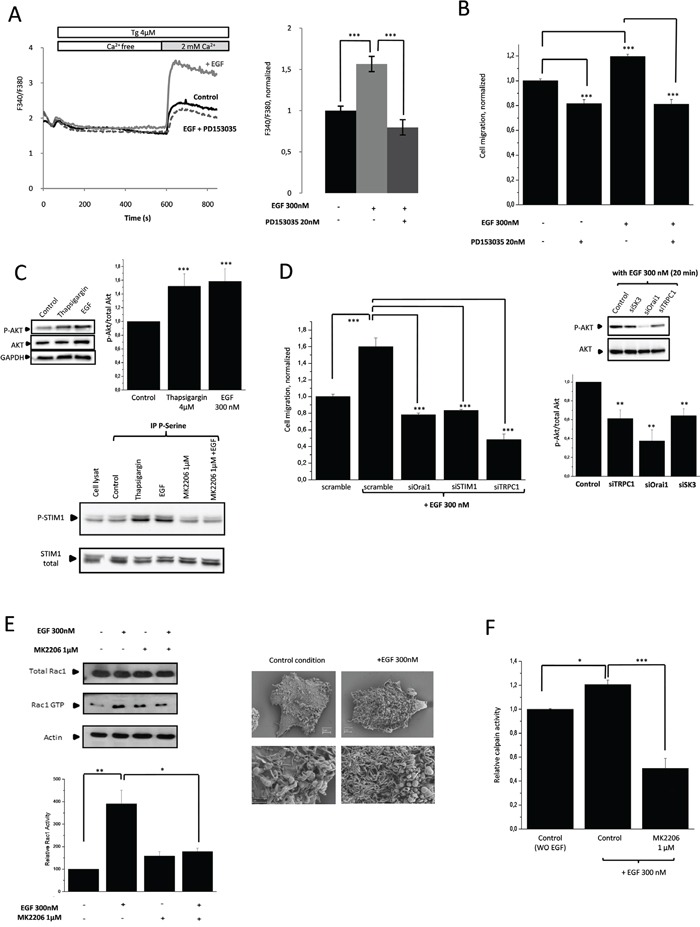
PI3K/Akt/Rac1 signaling pathway mediates EGF-induced SOCE dependent cell migration *via* P-STIM1 **A.** Increased of SOCE induced by EGF is inhibited by PD153035. Fluorescence measurement and relative fluorescence of Ca^2+^entry after intracellular calcium store depletion by Tg in cells treated for 20min with EGF +/− PD153035. Results are expressed as mean ± SEM. ***p<0.001, sample significantly different from control (N=4, Kruskal-Wallis test). **B.** Increased of cell migration induced by EGF is inhibited by a selective ATP competitive inhibitor of EGFR (PD153035). Histograms showing HCT-116 cell migration with or without treatment by EGF +/− PD153035. Results are expressed as mean ± SEM. ***p<0.001, sample significantly different from control (N=3, n=7, Kruskal-Wallis test). **C.** Upper panel, EGF treatment and depletion of intracellular calcium store by Tg increase P-Aktin HCT-116 cells. Immunoblots representing P-Akt and total Akt in cells treated or not with EGF or Tg for 20min. P-Akt levels (standardized based on total Akt) was determined by densitometry scanning to generate the values shown in the bar graph. Results are expressed as mean ± SEM. ***p<0.005, sample significantly different from control (N=3, Kruskal-Wallis test). Lower panel, increase of SOCE induces by EGF appears to be linked to STIM1 phosphorylation by Akt. HCT-116 cells are treated with EGF +/− MK2206 or Tg. Serine-phosphorylated proteins were immunoprecipitated, and the presence of STIM1 in the immunocomplexes was detected by western blotting. **D.** Left panel, Silencing of calcium channels partners prevents SOCE-dependent migration induced by EGF. Results are expressed as mean ± SEM. ***p<0.001, sample significantly different from control (N=2, n=6 Kruskal-Wallis test). Right panel, dissociation of the lipid-raft Orai1/TRPC1/SK3 by siRNA prevents P-AKT increase mediated by EGF. Immunoblots representing P-Akt and total Akt in cells transfected with siRNA for 24h and treated 20 min with EGF.P-Akt levels (standardized based on total Akt) was determined by densitometry scanning to generate the values shown in the bar graph. Results are expressed as mean ± SEM. **p<0.005, sample significantly different from control (N=3, Kruskal-Wallis test). **E.** Inhibition of Akt by MK2206 decreases Rac1 activity (Rac1 GTP) enhanced by EGF treatment. Left, Upper panel, Immunoblots representing Rac1 GTP and total Rac1 in cells treated or not with EGF for 20min in combination with MK2206. Lower panel, activatedRac1 levels (standardized based on total Rac1) was determined by densitometry scanning to generate the values shown in the bar graph. Results are expressed as mean ± SEM. *p<0.05 and **p<0.01, sample significantly different from control (N=6, Kruskal-Wallis test). Right panel, HCT-116 cells imaged, before and after EGF treatment, using scanning electron microscopy. EGF enhances lamellipodial formation. **F.** Calpain activity in HCT-116 cells. Inhibition of Akt by MK2206 decreases calpain activity after EGF treatment. Results are expressed as mean ± SEM. *p<0.05 or ***p<0.001, sample significantly different from control (N=4, Kruskal-Wallis test).

Since the PI3K/Akt pathway is one of the main signaling pathways downstream of EGFR [16], we examined the link between dysregulated EGFR signaling and cancer cell migration. In HCT-116 cells, EGF increased SOCE and cell migration and both effects are inhibited by PD153035, a potent inhibitor of EGFR (Figure 4A, 4B). Note that EGF and Tg can both activate Akt in this cell line Figure 4C, upper panel). Interestingly, STIM1 was also found to be phosphorylated following Tg and EGF stimulations. Moreover, Akt activation was necessary to mediate EGF-dependent effect, since inhibition of Akt prevented STIM1 phosphorylation subsequently to EGF treatment (Figure 4C, lower panel). Therefore, phosphorylation of STIM1 (i.e., activation) following EGF stimulation and Akt-mediated signaling, appears to be involved in the stimulation process of SOCE and the promoter of cell migration and it is mediated by the transfer of TRPC1/Orai1 into lipid-rafts in proximity to SK3. As shown in Figure 4D, down-regulation of TRPC1/Orai1/P-STIM1 prevented the increase in cell migration induced by EGF to approximately the same level observed with PD153035 (Figure 4B). Interestingly, this was associated with a decrease in P-Akt (Figure 4D) suggesting a relationship between enhancement of P-Akt and SOCE by EGF.

Additionally, we showed that Rac1 activation by EGF was prevented by addition of an Akt inhibitor (Figure 4E). This finding is in agreement with the downstream activation of Rac1 by PI3K/Akt pathway following EGF stimulation. According to the increase in cell migration induced by Rac1, EGF stimulation increased the lamellipodial formation (Figure 4E). Moreover, EGF activated calpain and this activation was prevented by Akt inhibition (Figure 4F). Accordingly, enhancement of cancer cell migration by EGF appears to be linked to both the activation of the PI3K/Akt/Rac1/calpain pathway and SOCE increase, which is dependent onSTIM1 phosphorylation upon activation of both Rac1 and Akt regulation.

### Anti-EGFR mAbs effects converge on SOCE-dependent PI3K/Akt pathway in K-Ras- mutated cancer cells migration

**Figure 5:**
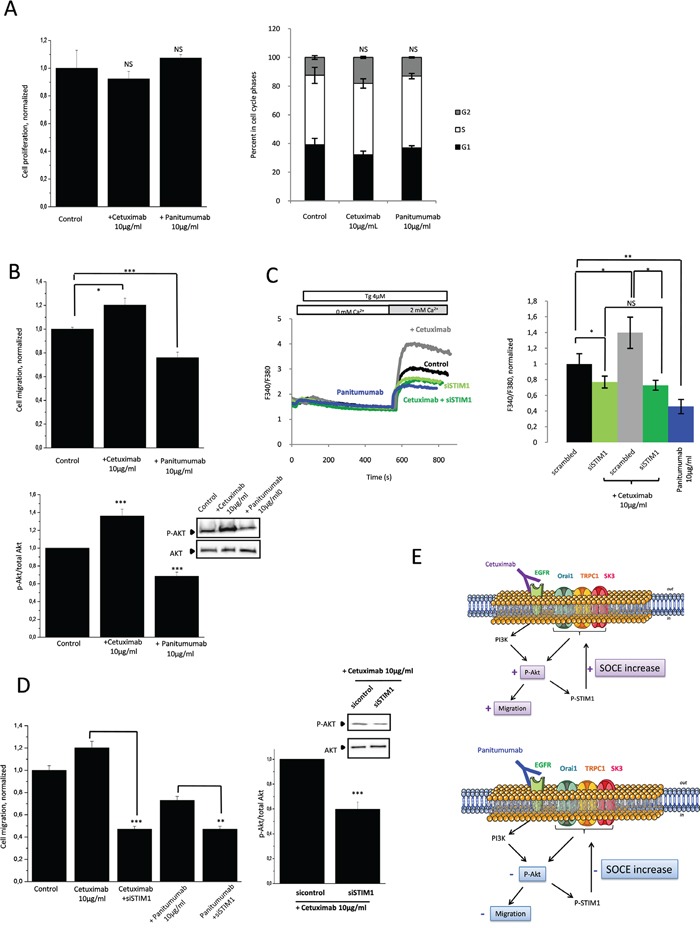
Anti-EGFR mAbs action converge with SOCE-dependent PI3K/Akt pathway involved in HCT-116 cell migration **A.** Effects of cetuximab and panitumumab on HCT-116 cell proliferation and cell cycle parameters. Left panel, cell viability of mAbs determined with the tetrazolium salt reduction method (MTT). HCT-116 cells were either treated with cetuximab or panitumumab 10μg/mL for 24 h. Right panel, histograms showing effects of cetuximab and panitumumab on cell cycle phases. Results are expressed as mean ± SEM. NS: sample not significantly different from control (N=3, Mann-Withney test). **B.** Effect of cetuximab and panitumumab on Akt-dependent cell migration. Upper panel, Histograms showing HCT-116 cell migration treated by cetuximab or panitumumab for 24h. Results are expressed as mean ± SEM. *p<0.05 or ***p<0.001, sample significantly different from control (N=3, Mann-Withney test). Lower panel, Immunoblots represent variation of P-Akt expression in HCT-116 cells treated with cetuximab or panitumumab for 24h. P-Akt levels (standardized based on total Akt) was determined by densitometry scanning to generate the values shown in the bar graph. Results are expressed as mean ± SEM. ***p<0.001, sample significantly different from control (N=3, Kruskal-Wallis test). **C.** Invalidation of STIM1, essential for SOCE, prevented the increase in calcium entry by cetuximab. Fluorescence measurement and relative fluorescence of Ca^2+^entry after intracellular calcium store depletion by Tg in cells treated for 24h +/− cetuximab and transfected or not with siSTIM1. Results are expressed as mean ± SEM. *p<0.05 or **p<0.01, sample significantly different from control (N=4, Kruskal-Wallis test). Panitumumab decreases SOCE in HCT-116. Fluorescence measurement and relative fluorescence of Ca^2+^entry after intracellular calcium store depletion by Tg in cells treated for 24h +/− panitumumab. Data are means ± SEM. **p<0.01, sample significantly different from control (N=7, Kruskal-Wallis test). **D.** Anti-EGFR mAbs actions converge with SOCE-dependent cell migration. Silencing of STIM inhibited the increased of migration enhances by cetuximab and amplifies the anti-migration effect of panitumumab. Left panel, Histograms showing cell migration in presence of cetuximab or panitumumab +/− siSTIM1. Results are expressed as mean ± SEM. ***p<0.001or **p<0.01, sample significantly different from control (N=3, n=9, Kruskal-Wallis test). Right panel, increased of Akt phosphorylation by cetuximab is inhibited by the down-regulation of STIM1. Immunoblots represent expression of P-Akt in HCT-116 cells treated with cetuximab +/− siSTIM1.P-Akt levels (standardized based on total Akt) was determined by densitometry scanning to generate the values shown in the bar graph. Results are expressed as mean ± SEM. ***p<0.001, sample significantly different from control (N=3, Kruskal-Wallis test) **E.** Proposed mechanism to explain the effects of cetuximab and panitumumabon EGFR signaling pathway and cell migration.

Due to the presence of K-Ras gene mutations, HCT-116 cells are resistant to the antiproliferative effects mediated by anti-EGFR therapies, cetuximab or panitumumab (Figure 5A). Nonetheless, the consequences of these mutations on Akt-dependent Ca^2+^ signaling are unknown. In Figure 5B, we show that cetuximab increased HCT-116 cells migration whereas panitumumab decreased it (upper panel). Akt phosphorylation was modified in a parallel manner: increased in presence of cetuximab and decreased by panitumumab (Figure 5B, lower panel). These effects correlated with SOCE activities as well (Figure 5C): cetuximab activated STIM1-dependent SOCE while panitumumab decreased it. Silencing of STIM1 abolished the modifying effect of these antibodies on cell migration (Figure 5D). Similary, silencing the Orai1/TRPC1/SK3 complex decreased the cetuximab-enhanced cell migration, and this observation was associated with a decrease in Akt phosphorylation (Supplementary Figure 1B). Our results showed that in K-Ras- and PI3K-mutated cancer cells, cetuximab and panitumumab used both the PI3K/Akt pathway to regulate in an opposite manner SOCE-dependent cancer cell migration (Figure 5E).

### Ohmline modifies the action of Anti-EGFR mAbs in mCRC

**Figure 6:**
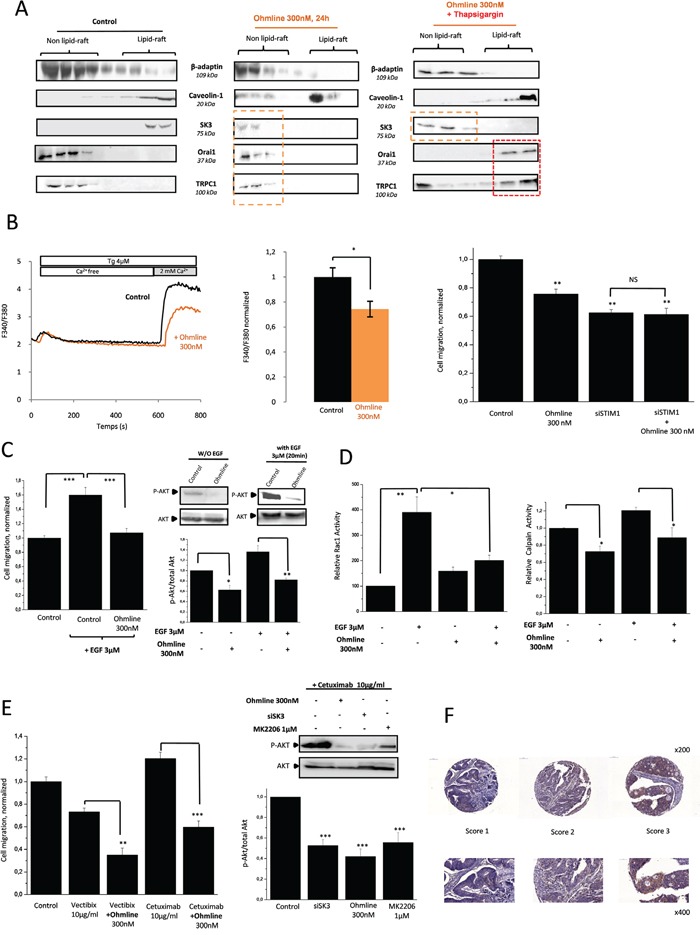
Ohmline as a new personalized treatment strategy to decrease P-Akt and therefore modulate the effects of Anti-EGFR mAbs **A.** Disrupting lipid-rafts with the alkyl-lipid Ohmline allows SK3 to re-translocate outside away from lipid-rafts without modifying the localization of calcium channels whereas the co-treatment Ohmline/Tg the translocation of calcium channels into lipid-raft. Immunoblots representing membrane fractionation, on a sucrose gradient, of cells treated with Ohmline alone (middle panel) or associated with 5μM Tg for 20 min (right panel). **B.** Left panel, Dissociation of the lipid-raft Orai1/TRPC1/SK3 channel complex by Ohmline decreased Ca^2+^entry evoked by Tg. Fluorescence measurement and relative fluorescence of Ca^2+^entry after intracellular calcium store depletion by Tg in cells treated 24h with Ohmline. Data represent means ± SEM. *p<0.05, sample significantly different from control (N=4, Mann–Whitney test). Right panel, Dissociation of the lipid-raft Orai1/TRPC1/SK3 channel complex by Ohmline decreased inhibits SOCE-dependent cell migration. Histograms showing HCT-116 cell migration treated with Ohmline +/− siSTIM1. The normalized cell number corresponds to the ratio of total number of migrating cells in presence of drugs/total number of migrating cells in control experiments. Results are expressed as mean ± SEM. **p<0.01, sample significantly different from control (N=3, n=9, Kruskal-Wallis test). **C.** Left panel, Dissociation of the lipid-raft Orai1/TRPC1/SK3 channel complex by Ohmline inhibits P-Akt-dependent cell migration enhanced by EGF. Results are expressed as mean ± SEM. ***p<0.001, sample significantly different from control (N=2, n=6 Kruskal-Wallis test). Right panel, Immunoblots show level of P-Akt in HCT-116 cells treated with Ohmline +/− EGF.P-Akt levels (standardized based on total Akt) was determined by densitometry scanning to generate the values shown in the bar graph. Results are expressed as mean ± SEM. *p< 0.5 and **p<0.01, sample significantly different from control (N=3, Kruskal-Wallis test). **D.** Dissociation of the lipid-raft Orai1/TRPC1/SK3 channel complexby Ohmline decrease Rac1 and calpain enhanced by EGF treatment. Left panel, activated Rac1 (standardized based on total Rac1) was determined by densitometric scanning to generate values shown in the bar graph. Results are expressed as mean ± SEM. *p<0.05 or **p<0.01, sample significantly different from control (N=6, Kruskal-Wallis test). Right panel, calpain activity results are expressed as mean ± SEM. *p<0.05 significantly different from control (N=4, Kruskal-Wallis test). **E.** Effect of cetuximab and panitumumab used in combination with Ohmline on cancer cell migration. Left panel, Histograms showing cell migration with cetuximab or panitumumab +/− Ohmline for 24h. Results are expressed as mean ± SEM. **p<0.1 and ***p<0.001, sample significantly different from control (N=3, n=9, Kruskal-Wallis test). Right panel, Immunoblots represent expression of P-Akt in HCT-116 cells treated with cetuximab +/− siSK3, Ohmline or MK2206.P-Akt levels (standardized based on total Akt) was determined by densitometry scanning to generate the values shown in the bar graph. Results are expressed as mean ± SEM. ***p<0.001, sample significantly different from control (N=3, Kruskal-Wallis test). **F.** Expression of SK3 protein in colon tissues. Representative images of cancer cells detected by SK3 immunostaining in primary tumors obtained from human colon cancer. A semiquantitative scoring was used to quantify SK3 levels on the basis of the data provided by the Human Protein Atlas (www.proteinatlas.org). Using this method, each staining/expression was scored as 0 (not detected), 1 (low intensity), 2 (medium intensity) or 3 (high intensity).

Ohmline treatment (Figure 6A, middle panel) induced a delocalization of SK3 channels from lipid-rafts along with a reduction of SOCE-dependent cell migration (Figure 6B). This effect was observed without affecting the translocation of the Orai1-TRPC1 channels, which remain in the lipid-rafts after Tg simulation (Figure 6A, right panel). These data support the enhancement of SOCE by SK3 channels when Orai1/TRPC1 channels are recruited in lipid-raft domains. Dissociation of the lipid-raft Orai1/TRPC1/SK3 complex by Ohmline prevented the increased cell migration (Figure 6C) and Akt phosphorylation mediated by EGF (Figure 6C, inset). Furthermore, the increase of Rac1-GTP and calpain activities by EGF was prevented by Ohmline treatment (Figure 6D). Accordingly, we show for the first time that an alkyl-lipid can modulate the PI3K/Akt/Rac1 pathway downstream of EGFR, which may be due to the dissociation of the lipid-raft Orai1/TRPC1/SK3 complex. In that case SK3 was translocated outside lipid-raft (Supplementary Figure 2). Furthermore, we hypothesized that cetuximab and panitumumab could be an effective treatment when combinated with Ohmline for a subset of mCRC patients carrying tumors with the K-Ras mutant. As shown in Figure 6E (left panel) co-treatment with Ohmline/mAbs decreased SOCE-dependent cell migration in both cases and this effect was correlated with the down-regulation of Akt phosphorylation (right panel). These results suggested that the combined inhibition of both EGFR signaling pathways associated with the selective translocation of SK3 outside lipid-rafts by Ohmline could represent a new approach to personalized therapeutic strategy designed to overcome the lack of inhibition of PI3K/Akt intracellular signaling when mAbs are used as single treatment agent. To assess the relevance of our findings to cancer patients, we assessed SK3 epithelial expression in clinical specimen of colon carcinoma. Most of the colon cancer samples (90%) obtained from primary tumors (167 of 184), showed SK3 positive epithelial staining (Figure 6F). In this perspective, it could be clinically relevant to target SK3 channel expression in patients whose tumors develop resistance to anti-EGFR therapies, in order to determine whether this adverse evolution could be reversed.

## DISCUSSION

Recently, human colon cancer cells have been shown to present an increase of SOCE, related to an increase in TRPC1, Orai1, Orai2, Orai3 and STIM1 expressions. These changes were correlated with increased cell proliferation, invasion and survival [17]. In this study, we showed that in HCT-116 cells, SK3 enhances SOCE mediated by the Orai1/TRPC1 channel complex. These results are different from previous reports mentioning that TRPC1 silencing does not inhibit SOCE in the colon cancer cell line HT29 [17]. We hypothesized that the involvement of TRPC1 in SOCE observed in HCT-116 cells results from the formation of an ion channel complex with SK3 whereas in HT29 cells, SK3 is not expressed and therefore does not participate to SOCE.

In contrast to epithelial breast cancer cells [12], STIM1 is involved in the formation of this complex in lipid-rafts where SK3 was localized. Formation of this complex leads to the formation of lipid-raft Cav-1/Orai1/TRPC1 complex cooperating with SK3 to promote SOCE-dependent cancer cell migration. STIM1 was defined as a phosphoprotein [18] with a cytosolic C-terminal domains containing serine residues targets for kinases following EGF stimulation [19]. More recently, it has been shown that serine residues of STIM1 are targets of extracellular-signal-regulated ERK1/2 and are involved in SOCE-dependent cancer cell migration [20, 21]. Here, we show that a mechanism of SOCE dependent on the activation of the PI3K/Akt pathway induced by EGF may be regulated by Akt-mediated phosphorylation of STIM1. This effect may play a role in the enhancement of the SOCE-dependent cell migration. Nevertheless, it remains to be explored whether STIM1 serine residues are targeted by Akt and whether SK3/Orai1/TRPC1 channels might be phosphorylated by Akt. In the PI3K/Akt signaling cascade, we showed that Rac1 basal activity can promote SOCE-dependent cell migration mediated by Ca^2+^-sensitive protease. In that case, calpain contributes to many pathways that control cell migration, such as cell spreading, membrane protrusion, chemotaxis, adhesion complex formation and membrane turnover [22]. M-calpain activity was shown to be significantly increased in colorectal adenocarcinoma [23]. In the integrin cascade, calpains act upstream of Rac1 and allow lamellipodia formation. Rac1 stimulates new actin polymerization and plays a key role in crosstalk between microtubules and actin filaments at the leading edge of migrating cells to promote lamellipodial formation.

**Figure 7:**
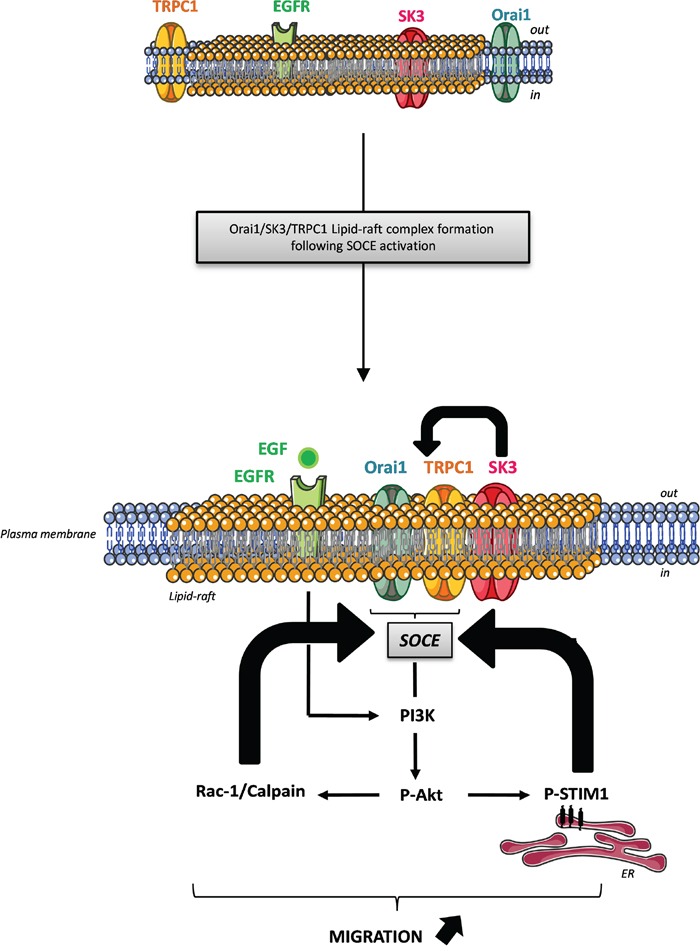
Proposed mechanism demonstrating the interaction between the lipid-raft associated Orai1/TRPC1/SK3 channel complex and EGFR signaling pathway in colon cancer cell migration This model suggests three positive feedback loops: 1) STIM1 following its phosphorylation by EGF stimulation and Akt, is the trigger of SOCE and promotes migration mediated by the translocation of TRPC1 and Orai1 into lipid rafts where SK3 is concentrated. 2) The Orai1/TRPC1/SK3 channel complex promotes SOCE, which enhances P-Akt leading to the phosphorylation of STIM1, that may promote SOCE and 3) P-Akt activates Rac1, which enhances SOCE and in turn P-Akt. These loops operated toward the same goal: enhancing SOCE and SK3-dependent cell migration.

Recently, Caveolin-1 has been described as a novel regulator of K-Ras-dependent migration of colon cancer cells [24]. Furthermore, caveolae-lipid-rafts provide signaling platforms for the signaling pathways activated by EGFR, which is frequently overexpressed in CRC [25] and seems to be associated with prognosis or survival [26, 27]. In the present study, we show that EGF, via binding to its receptor can activate SOCE through amplification of the action of basal P-Akt, leading to increased cancer cell migration. Our results suggest that amplification of SOCE induced by EGF could be due to the Akt-dependent phosphorylation of STIM1. Taken together these results suggest three positive feedback loops: 1) STIM1, after its phosphorylation by EGF and Akt, is stimulating SOCE and promotes migration mediated by the switch of TRPC1 and Orai1 into lipid-rafts where SK3 is concentrated. 2) SOCE may activate Akt, and SK3 appears to enhance SOCE: by hyperpolarizing the membrane, SK3 may increase the driving force for Ca^2+^and SOCE. 3) P-Akt activates Rac1/Calpain, which enhances SOCE and in turn P-Akt. These loops worked in the same fashion: enhancing SOCE and SK3-dependent cell migration (Figure 7).

Surprisingly, we showed that the two anti-EGFR mAbs, cetuximab and panitumumab, have an opposite effects on SOCE-dependent cell migration: while cetuximab induces SOCE-dependent cell migration, panitumumab decreases it. Furthermore, if the “EGF-like” effect of cetuximab on cell migration is abolished by the suppression of STIM1, this action enhances the reduction effect of panitumumab. This differential effect could be explained by the presence of Fc gamma receptor polymorphisms in colon cancer cells. These polymorphisms are known to modulate antibody-receptor engagement [28–32]. Indeed, mutational status has not been consistently correlated with mAbs efficacy in clinical trials [33, 34] suggesting that alternative molecular determinants of the response exist. Through the dissociation of SK3-Orai1 channel complex, we have shown that Ohmline inhibits breast cancer cell migration and metastases development [12, 35]. In HCT-116 cells, Ohmline also allows the translocation of SK3 outside lipid-raft and disrupted the positive feedback loop between SK3 and SOCs. This led to decrease SOCE, P-Akt, calpain and Rac1 activities under control condition or when EGF added. Interestingly, Ohmline was found to prevent the effects of cetuximab on cell migration through a decrease in P-Akt and to increase the inhibitory effect of panitumumab on cell migration. Importantly, this finding demonstrates for the first time that a lipid has the ability to modify anti-EGFR mAbs effects. PI3K/Akt inhibitors may be particularly useful in the case of CRC resistant to anti-EGFR mAbs therapy. This situation can occur when increased Akt signaling is associated with reduced sensitivity to cytotoxic agents or receptor tyrosine kinase inhibitors. Several studies have shown that dual treatment with selective inhibitors of PI3K/Akt and BRAF is associated with a synergistic tumor growth inhibition in mutated CRC cell lines presenting primary resistance to a BRAF inhibitor [36, 37]. To our knowledge, our study provides the first experimental evidence that an alkyl lipid may act as a modifier of the response to anti-EGFR mAbs in colorectal cancer cells: an alkyl-lipid can overcome their primary tumor resistance, where mAbs single treatment fails to inhibit PI3K/Akt intracellular signaling. In that regard, it may be clinically relevant to target the SK3/SOC/Akt signaling in mCRC patients. Nevertheless the capacity of Ohmline to inhibit PI3K/Akt in colon cancer cells mutated for PI3KCA and not expressing SK3 channels remains to be explored.

## MATERIALS AND METHODS

### Cell line and reagents

Colon cancer cell line HCT-116 was obtained from American Type Culture Collection and maintained in Opti-MEM supplemented with 10% fetal bovine serum, without antibiotics at 37°C in 95% (v/v) air/5% (v/v) CO_2_.

Epidermal Growth Factor human (EGF), LY294002, Apamin and Aminoethoxydiphenylborate (2-APB) are from Sigma-Aldrich. PD153035 and MK2206 are form Selleck Inc. Cetuximab is form MerckSerono. 1-Ohexadecyl-2-O-methyl-sn-glycero-3-lactose named Ohmline was synthetized as previously described [38].

### Intracellular Ca^2+^ measurements

Sticking cells were loaded in petri-dish for 45 min at 37°C with the ratiometric dye Fura2-AM (5 μM). Then, cells were trypsinized, washed with Opti-MEM^®^ Reduced Serum Medium, GlutaMax (Invitrogen) and centrifugated (x800g for 5min). Cells were suspended in PSS Ca^2+^-free solution and treated with thapsigargin (4μM) (T7458, Life-Technologies): to deplete the intracellular store depletion before injection of 2 mM of CaCl_2_. Fluorescence emission was measured at 510 nm using the monochromator of a spectrofluorimeter (Hitachi FL-2500, Ltd) with an excitation light at 340 and 380 nm. Maximum of fluorescence (Peak of calcium influx (F340/F380)) after injection of 2 mM of Ca^2+^ is measured and normalized to control condition. At least three independent experiments were performed.

### Membrane fractionation

Lipid-raft fractionation was carried out by using a detergent free, alkaline lysis method as described previously [12]. Caveolin-1 and β-adaptin were used as markers for the identification of caveolae and non lipid-rafts fractions, respectively.

### Immunohistochemistry

Tissue microarray (TMA) blocks were built on the basis of 200 formalin-fixed and paraffin-embedded colo-rectal samples (166 adenocarcinomas and 34 adenomas). Written informed consent was obtained from all patients and all samples were included in the registered tumor tissue collection n° DC-2008-214. Immunohistochemistry was performed on tissue section from the TMA blocks using SK3 antibody (HPA017990, Sigma Aldrich).

### Statistics

Statistical analyses have been performed using SigmaStat Software (Systat Software, Inc). Unless otherwise indicated, data were expressed as mean ± standard error of the mean (N, number of experiments and n, number of cells from independent experiments). For comparison between more than two means we used Kruskal-Wallis one-way analysis of variance followed by Dunn's or Dunnet's post hoc tests as appropriate. Comparisons between two means were made using Mann-Whitney. Differences were considered significant at p < 0.05.

### Transfection assay

Briefly, 2.5×10^5^ cells/well were plated in 6-well plates in Opti-MEM supplemented with 10% of FBS without antibiotics. Cells were incubated with a mix of siRNA and Lipofectamine in medium without serum for 6 hours. After incubation, an equal volume of medium with serum was added to each well. The siRNA sequences directed against Orai1 (sc-76001), TRPC1 (sc-42664) and STIM1 (sc-76589) were purchased from Santa Cruz Biotechnologies. For Control siRNA we used the following sequences: 5′CUGUAUCGAAUGUUAUGAGCC [34] 3′ and 5′GCUCAUAACAUUCGAUACAG [34] 3′ (Invitrogen). SK3 specific siRNA was designed asalready described (Potier *et al*., 2006). pEGFP Rac1N17 et pEGFP were transfected in HCT-116 cells with lipofectamine2000 (Invitrogen) according to the manufactory protocol. All siRNA transfections were performed for 48 hours.

### Electrophysiology

Electrophysiological recordings were performed in the whole cell configuration of the patch clamp technique as already described [38]. Patch pipettes (2.0-4.0 MΩ) were filled with a pipette solution contained (in mM): KCl 145, MgCl_2_ 1, Mg-ATP 1, HEPES 10, CaCl_2_ 0.87, EGTA 1, adjusted to pH 7.2 with KOH, pCa6 (final calcium concentration: 1 μM). Whole-cell macroscopic currents in HCT-116 cells were measured using a ramp protocol from −90 mV to +80 mV with a holding potential of −70 mV (2s duration; 2s intervals). In some experiments, currents were generated by stepwise 10 mV depolarizing pulses (2s duration; 4 sec intervals) from a constant holding potential of −40 mV and from potentials from −90mV up to +90 mV. Signals were filtered at 1 kHz and digitized at 5 kHz. The steady state current elicited (to build IV relation) at chosen membrane potential was calculated as the average of the current recorded during the latest 50 ms of the pulse. Current amplitudes of SK3 channels were analyzed at 0 mV to minimize chloride currents (E_Cl_^−^ = 0mV). Voltage clamp protocols were generated and the data captured with a computer using a Digidata 1200 interface, Axopatch 200B amplifier and pClamp9 software (Axon Instruments). The analysis was carried out using Clampfit 9(Axon Instruments), and Origin 6(Microcal Software) softwares.

### Cell proliferation and migration assays

Cell proliferation was determined using the tetrazolium salt reduction method (MTT), as described elsewhere [39]. Cells were seeded on 24-well plates at a density of 15,000 cells per well and measurements were performed in triplicate for 48 hours. Note that drugs concentration used in trans-well migration assays had no effect on cell proliferation/viability (48 hours).

Cell migration was analysed in 24-well plates receiving 8-μm pore size polyethylene terephthalate membrane cell culture inserts (Becton Dickinson), as previously described [40]. Briefly, 5×10^4^cells (wild-type cells or transfected cells for 24h) were seeded in the upper compartment with medium culture supplemented with 10% of FBS (± drugs/high external calcium concentration). The lower compartment was filled with medium culture supplemented with 10% FBS (± drugs/high external calcium concentration). Two-dimensional migration assays were performed without coating. After 24 h of migration and thus 48h of transfection, stationary cells were removed from the topside of the membrane, whereas migrated cells in the bottom side of the inserts were fixed, nuclei were stained and automatically counted [41]. At least three independent experiments were performed and each of them in triplicate.

### Western blot experiments

The antibodies used for western blot were the following: rabbit anti-Orai1 (H-46, Santa Cruz Biotech.), rabbit anti-Stim1 (GOK, BD), rabbit anti-GAPDH (D16H11, Cell Signaling Tech.), rabbit anti-SK3 (P0608, Sigma-Aldrich), rabbit anti-caveolin (D46G3, Cell Signaling Tech.), goat anti-β-adaptin (sc 6425, Santa Cruz Biotech.), mouse antiTRPC1 (E-6, Santa Cruz Biotech.), rabbit phospho-Akt (Ser473) and total Akt (Cell Signaling Tech.) and horseradish peroxidase conjugated anti-rabbit, anti-goat or anti-mouse (Jackson Immuno-Research Laboratories). For Co-immunoprecipitation we used Lightning-Link^®^ Horseradish Peroxidase (HRP) conjugation (Innova Biosciences) to label the rabbit anti-Orai1 (H-46, Santa Cruz Biotech.).

### Immunoprecipitation

For co-immunoprecipitation experiments, 500 μg of lysate were mixed into 500 μl of NET buffer (50 mMTris-HCl pH 7.4, 150 mMNaCl, 5 mM EDTA, 0.05% NP-40, pH 7.4).

Incubation was conducted overnight at 4°C with 2 μg of anti-STIM1 (sc-68897, Santa Cruz Biotech.) with continuous agitation. To precipitate the immune complexes, samples were incubated with 30 μl of protein A-sepharose (100 mg/mL) for 1 h at 4°C. Beadbound complexes were washed three times with NET buffer, eluted at 95°C for 5 min in the Laemmli sample buffer and analyzed by immunoblotting. As negative controls, 500 μg of lysate were immunoprecipitated with non-specific rabbit IgG antibodies. Total cell lysates and immunoprecipitated proteins were separated by SDS-PAGE using 9% polyacrylamide gels. Proteins were transferred and nitrocellulose membrane was then probed with primary antibodies.

### RacGTPase activity

#### Recombinant proteins

The GST-PAK-CRIB domain were obtained as pGEX-2 T fusion genes (gift of JG Collard, Netherlands Cancer Institute, Amsterdam, NL, USA) and produced as described [42]. Recombinant proteins were prepared as glutathione S-transferase fusion proteins in Escherichia coli (BL21 strain), purified using glutathione-sepharose beads (Amersham Pharmacia Biotech), and used as GST-fusion proteins.

#### Affinity binding assay

A total of 10^7^ HCT116 cells were washed twice in cold PBS and then lysed in 1ml of lysis buffer (TrisHCl 50mm pH 7.4, NaCl 100mm, MgCl2 2mm, 1% N P-40 (w/v), 10% glycerol, containing protease cocktail inhibitor 1X). Lysates were mixed into 50 μl of GST-fusion protein (GST-PAK-CD) corresponding to 5 mg of protein bound to glutathione-sepharose beads. Incubation was conducted at 4°C overnight. Bead-bound complexes were washed four times in lysis buffer, boiled in Laemmli sample buffer and fractionated by a 13% SDS–PAGE, followed by Western blotting. For the PAK-CD assay, the presence of Rac1 was revealed using anti-Rac1 antibodies clone 23A8 (05-389 Upstate, Millipore).

### Immunofluorescence

HCT-116 cells were fixed and permeabilized by methanol. Double staining was performed by incubation first with caveolin-1, and then with SK3 antibody (Anti-SK3-ATTO-594, Alomone Lab). Fluorescent images were captured with a JAI camera (model CV-M4+CL), with the use of an automated filter wheel coupled to a Leica DMRB fluorescence microscope (Leica Microsystems).

### Scanning electron microscopy

Briefly, cells were fixed and then fully dehydrated in a graded series of ethanol solutions and dried in hexamethyldisilazane (HMDS, Sigma, St-Louis, MO). Finally, the dry samples was sprinkled onto carbon disks and coated with 40 Å platinum, with a GATAN PECS 682 apparatus (Pleasanton, CA), before observation under a Zeiss Ultra plus FEG-SEM scanning electron microscope (Oberkochen, Germany).

### Cell cycle analysis

Cells were grown in the presence of mAbs (10μg/mL) for 24 h and treated with the Coulter DNA-Prep reagents kit (Beckman Coulter). The red fluorescence was analyzed by flow cytometry with a Coulter Epics Elite ESP flow cytometer (Beckman Coulter). Cell cycle distribution was analyzed with the Multicycle-AV software (Phoenix Flow Systems).

### Calpain activity assay

A calpain activity assay kit from Abcamwas used according to the manufacturer's recommendation. The fluorometric assay is based on the detection of cleavage of calpain substrate Ac-LLY-AFC. Fluorescence emission was measured at 505 nm using a microplate spectrofluorimeter (Molecular devices Spectra max 190, Ltd) with an excitation light at 400 nm.

## SUPPLEMENTARY FIGURES



## References

[1] Siegel R, Desantis C, Jemal A (2014). Colorectal cancer statistics, 2014. CA Cancer J Clin.

[2] Haggar FA, Boushey RP (2009). Colorectal cancer epidemiology: incidence, mortality, survival, and risk factors. Clinics in colon and rectal surgery.

[3] Cunningham D, Humblet Y, Siena S, Khayat D, Bleiberg H, Santoro A, Bets D, Mueser M, Harstrick A, Verslype C, Chau I, Van Cutsem E (2004). Cetuximab monotherapy and cetuximab plus irinotecan in irinotecan-refractory metastatic colorectal cancer. The New England journal of medicine.

[4] Van Cutsem E, Peeters M, Siena S, Humblet Y, Hendlisz A, Neyns B, Canon JL, Van Laethem JL, Maurel J, Richardson G, Wolf M, Amado RG (2007). Open-label phase III trial of panitumumab plus best supportive care compared with best supportive care alone in patients with chemotherapy-refractory metastatic colorectal cancer. J Clin Oncol.

[5] De Stefano A, Carlomagno C (2014). Beyond KRAS: Predictive factors of the efficacy of anti-EGFR monoclonal antibodies in the treatment of metastatic colorectal cancer. World journal of gastroenterology.

[6] Sanchez-Gonzalez P, Jellali K, Villalobo A (2010). Calmodulin-mediated regulation of the epidermal growth factor receptor. The FEBS journal.

[7] Enomoto K, Cossu MF, Maeno T, Edwards C, Oka T (1986). Involvement of the Ca2+-dependent K+ channel activity in the hyperpolarizing response induced by epidermal growth factor in mammary epithelial cells. FEBS letters.

[8] Peppelenbosch MP, Tertoolen LG, de Laat SW (1991). Epidermal growth factor-activated calcium and potassium channels. The Journal of biological chemistry.

[9] Roderick C, Reinach PS, Wang L, Lu L (2003). Modulation of rabbit corneal epithelial cell proliferation by growth factor-regulated K(+) channel activity. The Journal of membrane biology.

[10] Prevarskaya N, Skryma R, Shuba Y (2011). Calcium in tumour metastasis: new roles for known actors. Nat Rev Cancer.

[11] Gueguinou M, Chantome A, Fromont G, Bougnoux P, Vandier C, Potier-Cartereau M (2014). KCa and Ca(2+) channels: the complex thought. Biochimica et biophysica acta.

[12] Chantome A, Potier-Cartereau M, Clarysse L, Fromont G, Marionneau-Lambot S, Gueguinou M, Pages JC, Collin C, Oullier T, Girault A, Arbion F, Haelters JP, Jaffres PA, Pinault M, Besson P, Joulin V (2013). Pivotal role of the lipid Raft SK3-Orai1 complex in human cancer cell migration and bone metastases. Cancer research.

[13] Gueguinou M, Gambade A, Felix R, Chantome A, Fourbon Y, Bougnoux P, Weber G, Potier-Cartereau M, Vandier C (2015). Lipid rafts, KCa/ClCa/Ca channel complexes and EGFR signaling: Novel targets to reduce tumor development by lipids?. Biochimica et biophysica acta.

[14] Ong HL, Ambudkar IS (2011). The dynamic complexity of the TRPC1 channelosome. Channels Austin, Tex.

[15] Dart C (2010). Lipid microdomains and the regulation of ion channel function. The Journal of physiology.

[16] Safdari Y, Khalili M, Ebrahimzadeh MA, Yazdani Y, Farajnia S (2015). Natural inhibitors of PI3K/AKT signaling in breast cancer: Emphasis on newly-discovered molecular mechanisms of action. Pharmacol Res.

[17] Sobradillo D, Hernandez-Morales M, Ubierna D, Moyer MP, Nunez L, Villalobos C (2014). A reciprocal shift in transient receptor potential channel 1 (TRPC1) and stromal interaction molecule 2 (STIM2) contributes to Ca2+ remodeling and cancer hallmarks in colorectal carcinoma cells. The Journal of biological chemistry.

[18] Manji SS, Parker NJ, Williams RT, van Stekelenburg L, Pearson RB, Dziadek M, Smith PJ (2000). STIM1: a novel phosphoprotein located at the cell surface. Biochimica et biophysica acta.

[19] Olsen JV, Blagoev B, Gnad F, Macek B, Kumar C, Mortensen P, Mann M (2006). Global, in vivo, and site-specific phosphorylation dynamics in signaling networks. Cell.

[20] Casas-Rua V, Tomas-Martin P, Lopez-Guerrero AM, Alvarez IS, Pozo-Guisado E, Martin-Romero FJ (2015). STIM1 phosphorylation triggered by epidermal growth factor mediates cell migration. Biochimica et biophysica acta.

[21] Pozo-Guisado E, Campbell DG, Deak M, Alvarez-Barrientos A, Morrice NA, Alvarez IS, Alessi DR, Martin-Romero FJ (2010). Phosphorylation of STIM1 at ERK1/2 target sites modulates store-operated calcium entry. Journal of cell science.

[22] Franco SJ, Huttenlocher A (2005). Regulating cell migration: calpains make the cut. Journal of cell science.

[23] Lakshmikuttyamma A, Selvakumar P, Kanthan R, Kanthan SC, Sharma RK (2004). Overexpression of m-calpain in human colorectal adenocarcinomas. Cancer Epidemiol Biomarkers Prev.

[24] Basu Roy UK, Henkhaus RS, Loupakis F, Cremolini C, Gerner EW, Ignatenko NA (2013). Caveolin-1 is a novel regulator of K-RAS-dependent migration in colon carcinogenesis. International journal of cancer.

[25] Spano JP, Fagard R, Soria JC, Rixe O, Khayat D, Milano G (2005). Epidermal growth factor receptor signaling in colorectal cancer: preclinical data and therapeutic perspectives. Ann Oncol.

[26] Galizia G, Lieto E, Ferraraccio F, De Vita F, Castellano P, Orditura M, Imperatore V, La Mura A, La Manna G, Pinto M, Catalano G, Pignatelli C, Ciardiello F (2006). Prognostic significance of epidermal growth factor receptor expression in colon cancer patients undergoing curative surgery. Annals of surgical oncology.

[27] Nicholson RI, Gee JM, Harper ME (2001). EGFR and cancer prognosis. Eur J Cancer.

[28] Cartron G, Dacheux L, Salles G, Solal-Celigny P, Bardos P, Colombat P, Watier H (2002). Therapeutic activity of humanized anti-CD20 monoclonal antibody and polymorphism in IgG Fc receptor FcgammaRIIIa gene. Blood.

[29] Musolino A, Naldi N, Bortesi B, Pezzuolo D, Capelletti M, Missale G, Laccabue D, Zerbini A, Camisa R, Bisagni G, Neri TM, Ardizzoni A (2008). Immunoglobulin G fragment C receptor polymorphisms and clinical efficacy of trastuzumab-based therapy in patients with HER-2/neu-positive metastatic breast cancer. J Clin Oncol.

[30] Pander J, Gelderblom H, Antonini NF, Tol J, van Krieken JH, van der Straaten T, Punt CJ, Guchelaar HJ (2010). Correlation of FCGR3A and EGFR germline polymorphisms with the efficacy of cetuximab in KRAS wild-type metastatic colorectal cancer. Eur J Cancer.

[31] Zhang W, Gordon M, Schultheis AM, Yang DY, Nagashima F, Azuma M, Chang HM, Borucka E, Lurje G, Sherrod AE, Iqbal S, Groshen S, Lenz HJ (2007). FCGR2A and FCGR3A polymorphisms associated with clinical outcome of epidermal growth factor receptor expressing metastatic colorectal cancer patients treated with single-agent cetuximab. J Clin Oncol.

[32] Bibeau F, Lopez-Crapez E, Di Fiore F, Thezenas S, Ychou M, Blanchard F, Lamy A, Penault-Llorca F, Frebourg T, Michel P, Sabourin JC, Boissiere-Michot F (2009). Impact of Fc{gamma}RIIa-Fc{gamma}RIIIa polymorphisms and KRAS mutations on the clinical outcome of patients with metastatic colorectal cancer treated with cetuximab plus irinotecan. J Clin Oncol.

[33] Maughan TS, Adams RA, Smith CG, Meade AM, Seymour MT, Wilson RH, Idziaszczyk S, Harris R, Fisher D, Kenny SL, Kay E, Mitchell JK, Madi A, Jasani B, James MD, Bridgewater J (2009). Addition of cetuximab to oxaliplatin-based first-line combination chemotherapy for treatment of advanced colorectal cancer: results of the randomised phase 3 MRC COIN trial. Lancet.

[34] Moosmann N, von Weikersthal LF, Vehling-Kaiser U, Stauch M, Hass HG, Dietzfelbinger H, Oruzio D, Klein S, Zellmann K, Decker T, Schulze M, Abenhardt W, Puchtler G, Kappauf H, Mittermuller J, Haberl C (2011). Cetuximab plus capecitabine and irinotecan compared with cetuximab plus capecitabine and oxaliplatin as first-line treatment for patients with metastatic colorectal cancer: AIO KRK-0104—a randomized trial of the German AIO CRC study group. J Clin Oncol.

[35] Girault A, Haelters JP, Potier-Cartereau M, Chantome A, Jaffres PA, Bougnoux P, Joulin V, Vandier C (2012). Targeting SKCa channels in cancer: potential new therapeutic approaches. Current medicinal chemistry.

[36] Oikonomou E, Koc M, Sourkova V, Andera L, Pintzas A (2011). Selective BRAFV600E inhibitor PLX4720, requires TRAIL assistance to overcome oncogenic PIK3CA resistance. PloS one.

[37] Yang H, Higgins B, Kolinsky K, Packman K, Bradley WD, Lee RJ, Schostack K, Simcox ME, Kopetz S, Heimbrook D, Lestini B, Bollag G, Su F (2012). Antitumor activity of BRAF inhibitor vemurafenib in preclinical models of BRAF-mutant colorectal cancer. Cancer research.

[38] Girault A, Haelters JP, Potier-Cartereau M, Chantome A, Pinault M, Marionneau-Lambot S, Oullier T, Simon G, Couthon-Gourves H, Jaffres PA, Corbel B, Bougnoux P, Joulin V, Vandier C (2011). New alkyl-lipid blockers of SK3 channels reduce cancer cell migration and occurrence of metastasis. Current cancer drug targets.

[39] Potier M, Joulin V, Roger S, Besson P, Jourdan ML, Leguennec JY, Bougnoux P, Vandier C (2006). Identification of SK3 channel as a new mediator of breast cancer cell migration. Molecular cancer therapeutics.

[40] Chantome A, Girault A, Potier M, Collin C, Vaudin P, Pages JC, Vandier C, Joulin V (2009). KCa2. 3 channel-dependent hyperpolarization increases melanoma cell motility. Experimental cell research.

[41] Brouard T CA (2009). Automatic nuceli cell counting in low-resolution fluorescence images. Computational Vision and Medical Image Processing.

[42] Sander EE, van Delft S, ten Klooster JP, Reid T, van der Kammen RA, Michiels F, Collard JG (1998). Matrix-dependent Tiam1/Rac signaling in epithelial cells promotes either cell-cell adhesion or cell migration and is regulated by phosphatidylinositol 3-kinase. The Journal of cell biology.

