# Supplementary material for: SK3/TRPC1/Orai1 complex regulates SOCE-dependent colon cancer cell migration: a novel opportunity to modulate anti-EGFR mAb action by the alkyl-lipid Ohmline
Source: Oncotarget. 2016 Apr 18;7(24):36168–84. doi: 10.18632/oncotarget.8786 (PMC5094991; doi:10.18632/oncotarget.8786)
Supplement: Supplementary file 1 [file oncotarget-07-36168-s001.pdf]

SK3/TRPC1/Orai1 complex regulates SOCE-dependent colon cancer cell migration: a novel opportunity to modulate anti-EGFR mAb action by the alkyl-lipid Ohmline

SUPPLEMENTARY FIGURES

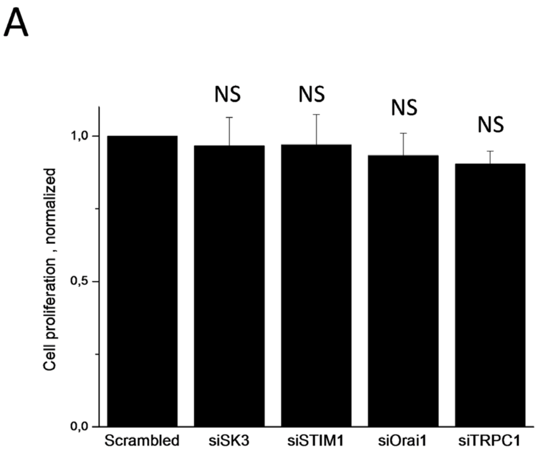

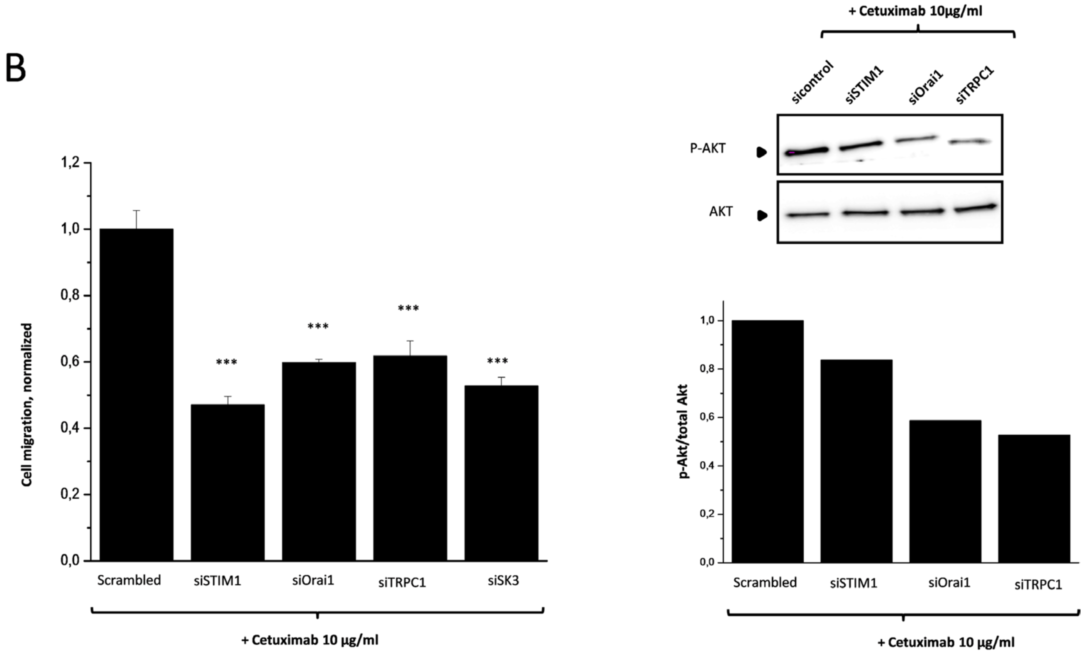

Supplementary Figure S1: A. Effects of siRNA directed against channel complex partners on HCT-116 cell proliferation. Cell viability determined with the tetrazolium salt reduction method (MTT). Histograms showing effects of sicontrol, siSK3, siOrai, siTRPC1 and siSTIM1 on HCT-116 cells viability for 48 h. Results are expressed as mean ± SEM. NS: sample not significantly different from control (N=3, Mann-Withney test). **B.** Silencing of SK3, Orai1, STIM1 and TRPC1 decreased cetuximab-dependent cell migration. Left panel, Histograms showing HCT-116 cells migration in control condition or after silencing of SK3, Orai1, STIM1 and TRPC1 for 48h. The normalized cell number corresponds to the ratio of total number of migrating cells in presence of drugs/total number of migrating cells in control experiments. Results are expressed as mean ± SEM. **p<0.01, sample significantly different from control (N=3, n=9, Kruskal-Wallis test). Right panel, Increase of Akt phosphorylation by cetuximab is inhibited by silencing of STIM1, Orai1, and TRPC1. Immunoblots represent expression of P-Akt in HCT-116 cells treated with cetuximab +/- siSTIM1, siOrai1, siTRPC1. siRNA against channels partners decrease Akt activation. P-Akt levels (standardized based on total Akt) was determined by densitometry scanning to generate the values shown in the bar graph. N=1. Immunoblots representing P-Akt and total Akt in cells under control condition and after transfection with siRNA for 24h.

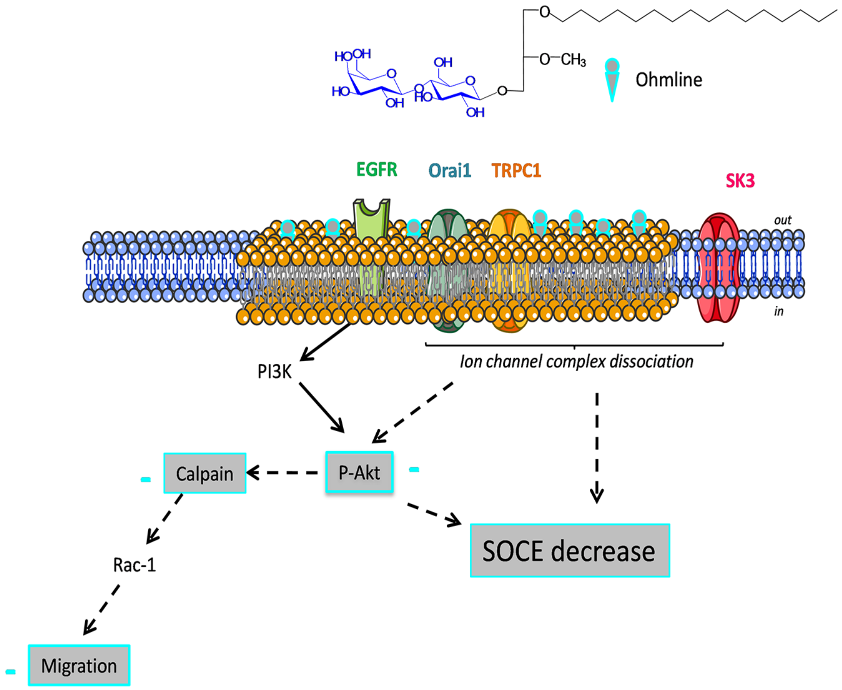

Supplementary Figure S2: Proposed mechanism between the lipid-raft Orai1/TRPC1/SK3 channel complex and Ohmline effect on EGFR signaling pathway in colon cancer cell migration. Disrupting lipid-rafts with the alkyl-lipid Ohmline allows SK3 to move away from lipid-rafts without modify localization of calcium channels and leads to a decrease of P-Akt-dependent cell migration.
